# An eScience-Bayes strategy for analyzing omics data

**DOI:** 10.1186/1471-2105-11-282

**Published:** 2010-05-26

**Authors:** Martin Eklund, Ola Spjuth, Jarl ES Wikberg

**Affiliations:** 1 Department of Pharmaceutical Biosciences, Uppsala University, P.O. Box 591, SE 751 24 Uppsala, Sweden

## Abstract

**Background:**

The omics fields promise to revolutionize our understanding of biology and biomedicine. However, their potential is compromised by the challenge to analyze the huge datasets produced. Analysis of omics data is plagued by the curse of dimensionality, resulting in imprecise estimates of model parameters and performance. Moreover, the integration of omics data with other data sources is difficult to shoehorn into classical statistical models. This has resulted in *ad hoc *approaches to address specific problems.

**Results:**

We present a general approach to omics data analysis that alleviates these problems. By combining eScience and Bayesian methods, we retrieve scientific information and data from multiple sources and coherently incorporate them into large models. These models improve the accuracy of predictions and offer new insights into the underlying mechanisms. This "eScience-Bayes" approach is demonstrated in two proof-of-principle applications, one for breast cancer prognosis prediction from transcriptomic data and one for protein-protein interaction studies based on proteomic data.

**Conclusions:**

Bayesian statistics provide the flexibility to tailor statistical models to the complex data structures in omics biology as well as permitting coherent integration of multiple data sources. However, Bayesian methods are in general computationally demanding and require specification of possibly thousands of prior distributions. eScience can help us overcome these difficulties. The eScience-Bayes thus approach permits us to fully leverage on the advantages of Bayesian methods, resulting in models with improved predictive performance that gives more information about the underlying biological system.

## Background

High-throughput experimental methods, including DNA and protein microarrays and other omics techniques, have become ubiquitous, indispensable tools in biology and biomedicine. The number of high-throughput technologies is constantly increasing. They provide the power to measure thousands of features of a biological system in a single experiment, and they have the potential to revolutionize our understanding of biology and medicine. However, the high expectations for omics methods have fallen short of realization, due to the challenges the data present for statistical modeling. Thus, the wealth of data produced is difficult to translate into concrete biological knowledge, new drugs, and clinical practices [1,2]. A recurring problem is that few experimental samples are generated relative to the number of model parameters [1]. This leads to imprecise parameter and performance estimates, and models prone to overfitting (the 'curse of dimensionality'). In fact, it is difficult to even obtain a trustworthy measure of *how *imprecise performance estimates are with standard techniques like confidence intervals based on holdout estimates in cross-validation or bootstrapping [3,4]. Another problem is that classical statistical models are too restrictive to account for the complexities of integrating omics data with previously acquired data. This has forced us to resort to *ad hoc *approaches for solving very specific problems [5-7]. Statistical approaches that use prior distributions over the model parameters are known as Bayesian methods [8,9]. The prior distributions summarize one's belief about the parameters before seeing the data. After performing an experiment, the parameters are updated to a posterior distribution according to Bayes' theorem:

The posterior distribution reflects the contribution of both our prior beliefs and the experimental data (through the likelihood function).

Bayesian methods have a number of advantages that allow us to address the problems inherent in omics data analysis. Firstly, they afford formal and coherent incorporation of prior information and integration of data sources. Apart from the philosophical appeal of being able to use all available information when analyzing a problem, this allows us to tackle the curse of dimensionality in two ways: (1) Prior information can be used to reduce model dimensionality with Bayesian variable selection and regularization, and (2) data from different research studies can be coherently combined to increase the number of available observations. Bayesian modeling thus allows us to "borrow information" across studies [8]. Secondly, Bayesian methods correctly summarize the model's predictive distribution [10]. Thus, we can obtain reliable Bayesian confidence intervals (often called credible intervals) of the estimates of model performance in small-sample problems; this avoids the problem of having to rely on imprecise confidence intervals based on holdout estimates from cross-validation or bootstrapping [4]. Thirdly, Bayesian methods may be applied to problems with structures too complex for classical statistical methods [11]. Bayesian models can be made increasingly elaborate to accommodate for the integration of prior information and omics data from multiple data sources.

However, the advantages of Bayesian modeling come at a price. We have to specify prior distributions for all parameters in a model. For large models, like those for analyzing omics data, it is unrealistic to expect practitioners to manually collect prior information for all individual parameters. In practice, this limits the powerful advantage of using available prior knowledge. In addition, Bayesian methods require numerical handling of analytically intractable integrals. This is typically performed with Markov chain Monte Carlo (MCMC) methods, which are computationally very expensive and therefore restricts the size of the modeling problems that we can address.

**Figure 1:**
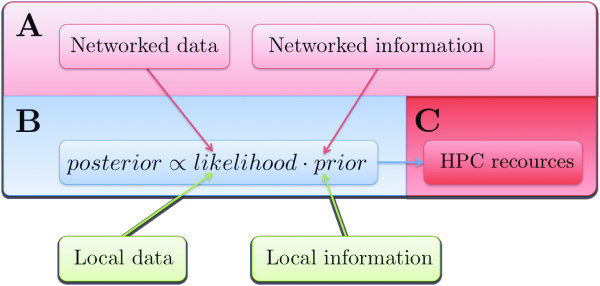
**The eScience-Bayes approach**. The red blocks represent the two aspects of eScience discussed in the main text: (light red) structured, federated, stored data and information that is retrievable with machines, and (dark red) networked computational power. The blue block represents the Bayesian part of the work flow: data setup and specification of the statistical model. The green blocks represent that locally produced data and information can be used together with the data and prior information retrieved from the Internet. **(A) **Interoperable machine-to-machine interactions, e.g. Web services, are employed to search the Internet for available information and data related to the biological or biomedical problem under study. **(B) **The biological or biomedical system is described in a Bayesian statistical model. The information collected in (**A**) is used to derive prior distributions of the parameters in the model, which summarize our *a priori *knowledge about the system. The data retrieved in (**A**) combined with any locally available data is used to update the prior distributions to a posterior distribution via the likelihood function (which represents the contribution of the data to the posterior distribution). **(C) **Fitting all but the simplest Bayesian models requires numerical integration, which is computationally highly demanding. Deploying the numerical integration on a HPC facility enables fitting large models in a manageable time.

Recent developments in distributed information and computational resources have given rise to the notion of eScience, i.e. computationally intensive science that is carried out in highly distributed network environments [12]. Advances in eScience can be roughly grouped into: (1) developments in semantic technologies (e.g. data standardization and ontologization) and methods for retrieving standardized data (e.g. Web services); and (2) construction of high-performance computing (HPC) facilities and development of middleware for using the HPCs. These two aspects of eScience equip us for efficient use of the Bayesian approach to omics data analysis. Standardized data and Web services allow us to use machines to harvest the Internet for information to use in prior distribution specifications, and HPC resources provide the computational power required to fit large Bayesian models to high-throughput data (Fig. 1). In this paper we demonstrate how eScience permit us to leverage on the advantages of Bayesian analysis when modeling omics data. We show by two examples that the approach can improve predictive performance, and that it permits a deeper analysis of the data by accommodating more complex model structures.

## Results

### Transcriptomics data: Predicting distant metastasis development in breast cancer patients

Microarray gene expression profiling has shown promise for supporting the prognostication of breast cancer patients based on the expression pattern of specific gene sets ('signatures'). A number of studies have reported different signatures [13-16]. However, concerns have been raised against the prognostic capacity of these signatures when applied to new data [17,18]. These concerns include the following:

1. Signatures from different studies share almost no genes, which indicates that the signatures depend to a large degree on the datasets rather than being prognostic for breast cancer [17]. In fact, Michiels *et al*. [18] showed that, even *within *a study, the subset of patients used for signature derivation strongly influenced which genes were selected.

2. The reported performance estimates of the signatures have been questioned [18]; this suggests that worse performance is likely to be obtained when applied to new data. Also, the confidence intervals of the performance estimates (if reported at all) have been disputed [4,18]. This makes it difficult to assess the level of accuracy in the reported results.

**Figure 2:**
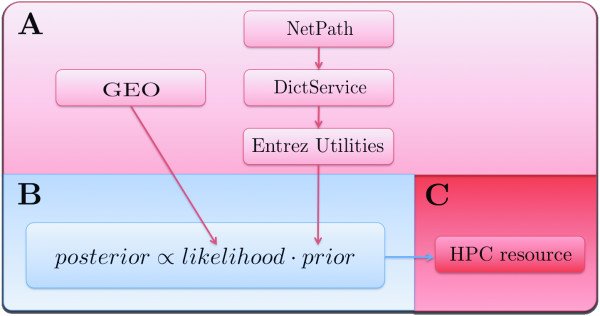
**eScience-Bayes applied to microarray gene expression data**. Illustration of using the eScience-Bayes approach to model time to development of distant metastases in breast cancer patients from microarray gene expression data. (Color-coding is as in Fig. 1; the green blocks in Fig. 1 are not included since we did not use any locally produced data or information.) **(A) **We downloaded five breast cancer gene expression datasets and their associated clinical data using the Gene Expression Omnibus (GEO) Web service. For trustworthy prior information about genes with altered transcriptional regulation in breast cancer patients, we used three Web services in conjunction: NetPath, DictService and Entrez Utilities. This gave us a list of integers that represented the number of times a cancer-pathway regulated gene on the HG-U133A array was mentioned in PubMed articles together with breast cancer. **(B) **We used the prior information derived in (**A**) to restrict the dimensionality of Models I and II by Bayesian variable selection. We did this by deriving prior distributions based on the assumption that the probability of a gene being related to breast cancer was reflected in the number of times a gene reported in NetPath was mentioned in combination with breast cancer in PubMed articles. **(C) **The models were fit by performing calculations a HPC resource.

**Figure 3:**
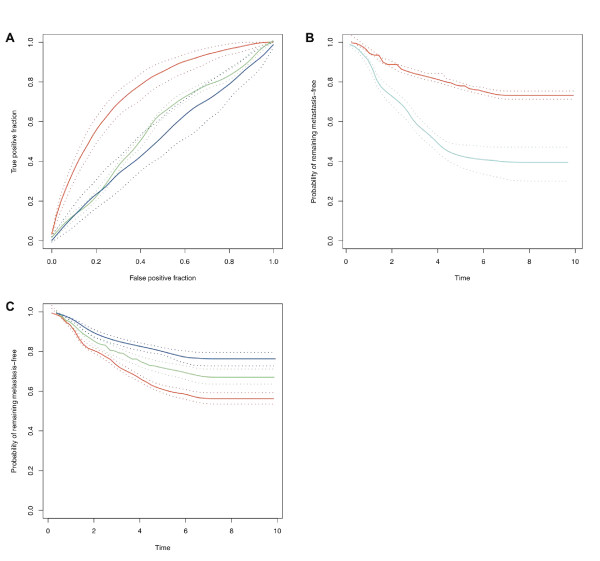
**Model I and II results**. ROC curves and Kaplan-Meier (KM) curves produced with Model I and II (data from Desmedt *et al*. [20], Miller *et al*. [14], and Sotiriou *et al*. [15] was used for training, and the data from Wang *et al*. [13] and Pawitan *et al*. [16] for testing). Solid lines show the mode of the curves? distributions; dotted lines show the 95% Bayesian confidence intervals. **(A) **ROC curves for Model I. Red and green lines represent Model I results after training on multiple datasets with informative and noninformative priors, receptively. Blue lines represent Model I after training on a single dataset. The area under the curves (AUC) are 0.76 (0.71; 0.80), 0.57 (0.55; 0.58), and 0.51 (0.44; 0.60), respectively (numbers in parentheses show the Bayesian confidence intervals). The predictive performance of Model I trained with multiple datasets and informative priors was found to be significantly better than when using noninformative priors and with only a single dataset for training (Bayesian p-value < 0.0005 in both cases). **(B) **KM curves [44] for patients predicted by Model I (at 80% specificity) indicate the probability that patients develop distant metastases before (red) and after (cyan) 5 years. **(C) **Survival times predicted by Model II. The KM curves show survival of patients belonging to the predicted percentiles 0-33, 34-66, 67-100 (red, green, and red curves, respectively). The difference between the three groups is significant (Bayesian P-value < 0.003).

In fact, both these concerns arise due to the small sample sizes typically used in microarray studies relative to the number of profiled genes (i.e. the curse of dimensionality). We employed the eScience-Bayes strategy described in Fig. 1 to address this problem. In accordance with the practice in most previous articles, we modeled the development of distant metastases within five years. To increase the sample size, we used the Gene Expression Omnibus (GEO) Web service [19] to collect data from five previously published breast cancer studies [13-16,20]. To reduce the model dimensionality we employed a Bayesian variable selection procedure, giving higher prior probabilities of including a variable (gene) the higher our prior belief that the gene affects development of distant metastases. The prior belief for each gene to affect development of distant metastases was based on information retrieved by connecting three Web services: NetPath http://www.netpath.org/, DictService http://services.aonaware.com/ and Entrez Utilities http://eutils.ncbi.nlm.nih.gov/entrez/eutils/soap/v2.0/DOC/esoap_help.html. NetPath is a curated resource of genes reported to be transcriptionally regulated by cancer-signalling pathways. All genes on the HG-U133A array listed in NetPath were used to text mine PubMed for all free fulltext articles where the gene names were mentioned in combination with breast cancer. To reduce the number of spurious hits, we discarded genes with names that represent English words by using the dictionary definition Web service DictService. This gave us a list of integers, associated with the genes on the HG-U133A array representing the number of times a cancer-pathway regulated gene is mentioned together with breast cancer in the literature (all genes not present in NetPath were assigned the number 0). These informative prior distributions were thus based on the assumption that the probability of a gene being related to breast cancer was reflected in the number of times a gene reported in NetPath was mentioned in combination with breast cancer in PubMed articles. A work flow of the procedure is shown in Fig. 2 (further details are given in Methods). The five datasets and the prior distributions were incorporated in a multilevel Bayesian probit model (**Model I**, see Methods). To assess the discriminative power of Model I, we fitted it five times; each time using three of the datasets as training sets and the remaining two datasets as independent testsets (Fig. 3a, red curve, and b; Additional file 1, Fig. S1 and S2; Table S1). It may be noted that Model I achieved high prediction accuracy on independent test data produced in different research groups. To the best of our knowledge, this is the first time that a modeling approach has demonstrated consistently good predictive ability across multiple independent testsets for predicting breast cancer metastasis development from gene expression data.

To validate the value of using prior information, we tested Model I with non-informative prior distributions (i.e. we assigned equal prior probability for all variables to be included in the variable selection; see Methods for further details) and compared the results to those obtained with informative priors (Fig. 3a, green curve; Additional file 1, Fig. S1, green curves; Table S1). This clearly showed that the use of relevant prior information significantly improves the classification accuracy of Model I (Bayesian P-value < 0.001, related to the curves in Fig. 3a). The dramatic improvement was due to the fact that the prior information focused the model to include relevant genes in the signature, thus reducing the risk of detecting spurious relationships and overfitting the model. Analogously, the use of prior information enforced consistency in signature selection across disparate datasets: 15.2% of the genes were selected all five times Model I with informative priors was fit, which may be compared to 1% when noninformative priors were used, and 0% among the original signatures. The median of the pairwise overlap between two different model fits was 27.5%, compared to 3% among the original signatures and 12% reported in Chuang *et al*. [6].

We also assessed the advantage of using multiple datasets by using only single datasets for training Model I (with informative priors); results are shown in Fig. 3a (blue curve), Additional file 1, Fig. S1 (blue curves) and Table S1. A significant improvement was found when multiple datasets were used for model training compared to when only a single dataset was used (Bayesian P-value < 0.001, related to the curves in Fig. 3a). In a final comparison, we modified Model I to use only the expression data that corresponded to the respective gene signatures derived in Wang *et al*. [13], Miller *et al*. [14], Sotiriou *et al*. [15], and Pawitan *et al*. [16], together with noninformative priors (only the genes printed on the HG-U133A were used for the Miller *et al*. [14] and Pawitan *et al*. [16] signatures). Again, we found that Model I (with informative priors) performed significantly better than when the signatures from Wang *et al*. [13], Miller *et al*. [14], Sotiriou *et al*. [15], and Pawitan *et al*. [16] were used. Results are displayed in Additional file 1, Fig. S4 and Table S1. To derive a "final" gene signature, we fitted Model I using all five datasets and informative priors. Some genes were included in this signature that *a priori *had been regarded as non-relevant to breast cancer; these genes were strongly supported by the data and may be of interest for future experimental investigations. The genes included in the signature are shown in Additional file 1, Table S2, stratified into genes that were relevant *a priori *as well as *a posteriori*, and genes that were regarded non-relevant *a priori *but relevant *a posteriori*.

In order to further demonstrate the eScience-Bayes approach, we used it to create a model that predicted the probability of distant metastasis development as a function of time. This model would provide finer granularity in the prognosis predictions of future patients compared to the current practice of stratifying patients into only two classes (metastases or no metastases within five years). Patient stratification into two classes discards information by discretizing a continuous variable (time to development of distant metastasis) into an arbitrarily defined binary variable (yes/no development of distant metastasis within five years). Because not all patients had developed distant metastases before the last follow-up in the studies, this required a consideration of the censoring of the data (Methods). We created a multilevel Bayesian accelerated failure time (AFT) (Methods; Sha *et al*. [21]) model with the expression data as explanatory variables and the informative prior distributions (**Model II**, see Methods for details). Analogously to the assessment of Models I, we assessed Model II by fitting it five times, each time using three of the datasets as a training set and the remaining two datasets as test sets to assess the performance of Model II. The results implied that we indeed could provide more fine-grained prognoses predictions, compared to the binary classification afforded with Model I (Fig. 3c).

In this example, we provided evidence for that adopting the eScience-Bayes approach can significantly improve consistency in gene signature selection and in the predictive performance of the constructed models. In contrast to previous studies, our results were presented together with Bayesian confidence intervals, which permit assessment of the level of accuracy in the results [4].

### Proteomics data: Analyzing PDZ domain-peptide interactions

**Figure 4:**
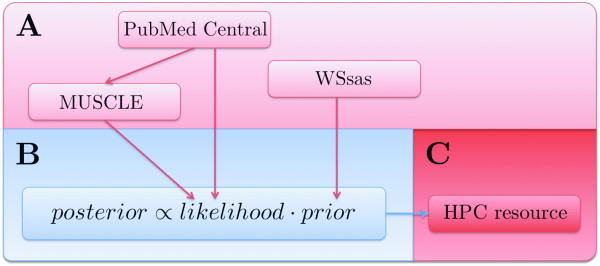
**eScience-Bayes applied to protein-protein interaction data**. Modeling of the PDZ domain-peptide interaction data with the eScience-Bayes approach. (Color-coding is as in Fig. 1.) **(A) **All PDZ and peptide sequences and interaction data were retrieved from the supplementary material of Chen *et al*. [23] via PubMed Central. The PDZ sequences were aligned using the MUSCLE Web service. Prior information was retrieved using the SAS Web service. SAS was used to determine the number of contacts made between PDZ domain residues and ligand residues from the 3D complexes available in the Protein Data Bank. **(B) **The aligned PDZ domain and peptide sequences were characterized by numbers capturing their physicochemical properties. The characterizations of the PDZ domain-peptide interactions were correlated to a 1 or a 0 in a binary response variable, which indicated whether a PDZ domain and a peptide could bind or not to each other. Analogous to the breast cancer demonstration, we reduce the dimensionality of the model guided by the prior information derived in (**A**) (see Methods). **(C) **The models were fit on an HPC resource.

**Figure 5:**
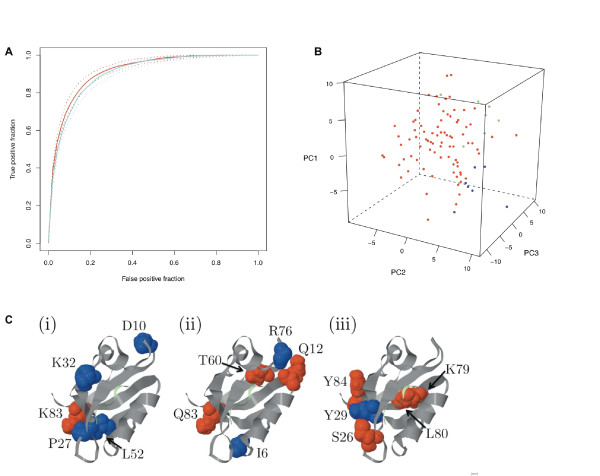
**Model III results**. **(A) **The ROC curves for Model III; the red and blue lines show the results when the prior information was or was not used, respectively. Solid lines denote the mode of the ROC curve distribution, and the dotted lines the 95% Bayesian confidence intervals. The area under the curves (AUC) are 0.92 (0.89;0.93) (red) and 0.90 (0.87;0.92) (blue). **(B) **Trends in differences between different PDZ domains were obtained by allowing the regression coefficients to vary with PDZ domains, and then subjecting the resulting matrix of regression coefficients to a principal component analysis (PCA) and plotting the PDZ domains in the space of the first three principal components. It should be noted that the regression coefficients are evenly distributed, which supports the relaxation of the canonical PDZ classes proposed in e.g. Stiffler *et al*. [22] and Tonikian *et al*. [37]. The regression coefficients from interactions in *C. elegans *(green dots) and *D. melanogaster *(blue dots) cluster together in the outskirts of the mouse interactions (red dots).**(C) **Differences in predicted networks between different PDZ domains. Five residues (spacefilled) located outside the binding pocket that were estimated with Model III to be allosterically linked to residues in the binding peptide (light green) in representative PDZ domains: (i) mouse *α*1-syntrophin(1/1), (ii) *D. melanogaster *Lap4(2/4), and (iii) *C. elegans *Lin7(1/1). The network residues are color coded according to the physicochemical property that is estimated to be important for the network: blue = size; red = polarity.

PDZ domains mediate protein-protein interactions and have been extensively studied over the last fifteen years. Recently, a large-scale PDZ domain-peptide interaction dataset was published in Stiffler *et al*. [22] and follow-up studies were later described in Chen *et al*. [23]. The combined data from these two publications comprise 2306 observations of interactions between peptides and PDZ domains from mouse, *C. elegans*, and *D. melanogaster*. We reanalyzed these data using the eScience-Bayes approach. To this end, we modeled the PDZ domain-peptide interactions using a multilevel Bayesian probit model with physicochemical properties of the PDZ domains and peptides as explanatory variables and a binary variable as response, indicating whether or not a PDZ domain and a peptide bound to each other (**Model III**). We derived informative prior distributions from 3D structural information using the Sequence Annotated by Structure [24] (SAS), a tool for annotating a protein sequence with structural information based on all solved 3D structures of the proteins in the Protein Data Bank [25] (PDB). Using the SAS Web service [26] (WSsas) we estiamted the number of contacts made between residues in each PDZ domain and in the peptide ligands. Similarly to the breast cancer demonstration, we used the informative prior distributions to "guide" the variable selection in order to reduce the dimensionality of the model based on the assumption that the importance of a PDZ domain residue for the interaction is reflected in the number of contacts it makes with the ligand (see Methods for details). Fig. 4 shows the work flow for the prior elicitation and modeling. Fig. 5a summarizes the predictive performance of Model III in a receiver operating characteristics (ROC, see Methods) curve based on a 10-fold cross-validation, and shows a comparison with the predictive performance when noninformative prior distributions were used (i.e. equal prior probability for all variables to be included in the variable selection; see Methods). We observed a nonsignificant (Bayesian P-value = 0.11) improvement in the predictive performance with informative compared to noninformative priors (Fig. 5a).

In this example, the main advantage of adopting an eScience-Bayes approach was the increase in information that we obtained. For example, Model III enabled an analysis of the differences between PDZ domains. By allowing the regression coefficients to vary between PDZ domains (Methods), we found differences in the estimated nonzero coefficients for different PDZ domains (Fig. 5b). This suggests that there may be variations among the PDZ domains in the networks of residues that are important for interacting with ligands [27]. Interestingly, some of these residues are located far away from the binding site. Although the tertiary structures of PDZ domains are very similar, their primary structures vary substantially [28]. This may cause differences in the intramolecular interactions within different PDZ domains. Networks of such intramolecular interaction have previously been reported for PDZ domains [28,29]. The results from studies by Gianni *et al*. [29] and Chi *et al*. [30] support the notion that the networks may differ among PDZ domains; for example, a network present in a PDZ domain from mouse tyrosine phosphatase BL was not found in the human PSD-95 PDZ3. The eScience-Bayes approach allowed analysis of these differences across a large set of PDZ domains.

Model III further indicated that the PDZ domains from *C. elegans *and *D. melanogaster *may form two clusters (Fig. 5b). Given the small datasets from *C. elegans *and *D. melanogaster*, which contain only seven PDZ domains each (Fig. 4), the clustering might be a sampling or an experimental artifact. Nevertheless, it is tantalizing to hypothesize that there are general trends in the intramolecular interaction networks in PDZ domains that differ between species. These observed differences are further illustrated in Fig 5c, where five residues, estimated to be allosterically linked to residues in the binding peptide, are shown in one representative PDZ domain from mouse, *C. elegans*, and *D. melanogaster*. As shown in the figure, different amino acids, with different positions and physicochemical properties, are estimated to be allosterically coupled to the binding peptide in the three different PDZ domains.

This example shows that an eScience-Bayes approach to modeling omics data can straightforwardly accommodate grouping structures in the data. This enables the analysis to reveal tentative differences among PDZ domains that allowed the inference of putative allosteric networks.

## Discussion and Conclusions

In a recent Nature Horizons article [12], Cambridge chemistry professor Peter Murray-Rust sketched an eScience world where the answer to any question is at the fingertips of every person; the complexity of the question and the size of the data to analyze do not matter. Computers perform all trivial and time-consuming tasks, like searching through millions of research articles or performing massive algorithmic calculations. In this proof-of-principle paper we have shown that some aspects of this vision in fact have been realized: machines can via Web services be used to retrieve background information that together with multiple data sources (produced locally or retrieved from the Internet) can be coherently exploited in a Bayesian framework to create complex models by employment of HPC resources, thus allowing us to provide answers to biologically relevant questions. This *modus operandi *contrasts to non-Bayesian approaches for merging multiple omics datasets and for integrating omics data with prior information, which to a large degree have relied on *ad hoc *methods for solving specific problems (see e.g. Kutalik *et al*. [5], Chuang *et al*. [6], and Xu *et al*. [7]). Bayesian statistics provide the flexibility to tailor statistical models to complex data structures, and can simultaneously accommodate prior information. The rather complex models described here would be diffcult to fit using classical statistics. We demonstrated the value of the Bayesian approach with the quite drastic improvements in prediction accuracy in the breast cancer example, and the enhanced depth of the data analysis in the PDZ domain-peptide interaction example. However, presently the eScience-Bayes approach is not easy to apply because it requires manual procurement of suitable information resources and Web services, and it requires programming skills to implement the Web service clients, parse the output, and connect them together. Moreover, the eScience-Bayes approach requires implementation of MCMC-algorithms to fit Bayesian models and knowledge of middleware and non-user-friendly interfaces for deployment of the MCMC-algorithms on HPC facilities. However, the most crucial difficulty is the prior elicitation process. Since the prior distributions are central in the Bayesian paradigm, a key aspect of applying the eScienceBayes approach is to summarize retrieved prior knowledge as distributions. In both the breast cancer and PDZ domain demonstrations we used prior information quantified as integers to represent our *a priori *belief of the independent variables relevance for modeling the response. Although care was taken to design the prior distributions in a sensible way, the prior specifications reflects a somewhat arbitrary step in the work flow, and there is presently no clear way to standardize prior elicitation and make the process transparent, exchangeable, and completely general. Related to this difficulty, another problem is that useful data and prior information in today's scientific practice are lost in objects that are inaccessible to computers, such as figures, tables, and summary statistics.

Thus, there is a distinct need for a standardized system of technologies and tools for working efficiently with computer representations of biological entities, data, information, probabilities, and statistical models - both locally and on the network. Great efforts are indeed being made in this direction. These include the increasing degrees of semantically annotated data that are deposited in public repositories in machine readable formats [31], new Web service protocols that allow for service discovery [32], semantic web technologies to adhere information with probability distributions that can be directly used as priors in Bayesian statistical models [33], and high-level languages for specifying fast implementations of MCMC algorithms [34]. Moreover, graphical workbenches, like Bioclipse [35], aim to handle all these tasks from a single point of entry. When these technologies and software have matured, it will be straightforward to adopt an eScience-Bayes approach to virtually any biological or clinical question. It would then be easy to obtain prior information and data by performing semantic queries, for example, "give me all breast cancer susceptibility genes and the probability distributions describing their association with increasing risk of distant metastasis development, together with all breast cancer Affymetrix HG-U133A datasets where distant metastasis development was the clinical end-point". The retrieved priors and data could then be used *directly *in a Bayesian statistical model specified in a high-level language and converted to an algorithmic fitting process deployed on an HPC facility. Finally, the results could be summarized graphically to increase the likelihood of making correct clinical prognostic and treatment decisions.

## Methods

### Transcriptomics data: Predicting distant metastasis development in breast cancer patients

The datasets with accession numbers GSE2034, GSE7390, GSE4922, GSE2990, and GSE1456 were retrieved with the GEO Web service [19]. The datasets were originally published in Wang *et al*. [13], Desmedt *et al*. [20], Miller *et al*. [14], Sotiriou *et al*. [15], and Pawitan *et al*. [16], and we hence denote them by (**X**, **t**, ***γ***)_*ρ *_= (**X**_*ρ*_, **t**_*ρ*_, ***γ***_*ρ*_), *ρ *∈ {*W, D, M, S, P*}. **X **represents gene expression measurements, **t **the time to development of distant metastses, and ***γ ***is a binary variable indicating censoring. All five datasets were generated with the Affymetrix HG-U133A platform and contained measurements of 22283 probes in 286, 198, 166, 189, and 159 patients, respectively (after removal of 85 replicate patients in the (**X**, **t**, ***γ***)_*M *_and (**X**, **t**, ***γ***)_*S *_datasets). The patients in the Miller *et al*. [14] and the Pawitan *et al*. [16] studies were profiled using both the Affymetrix HG-U133A and HG-U133B arrays; we here used only the data from the HG-U133A array. The columns in each **X**_*ρ *_were mean centered and scaled to unit variance.

We define *g*_*j*_, *j *= 1,...,22283, to be the gene names of the probes on the Affymetrix HG-U133A array, and  to be the set of gene names reported in NetPath as being transcriptionally regulated by cancer pathways, and  to be the set of words in the English language (Fig. 2). Further, set *h*_*j*_, *j *= 1,....,22283, to be the number of free, fulltext articles in PubMed where the gene name *g*_*j *_was mentioned. Finally, let *k*_*j*_, *j *= 1,...,22283, be defined according to:(1)

#### Model I

We modeled whether development of distant metastases occurred within five years using a multilevel Bayesian probit model according to:(2)

where *Pr *denotes probability, and Φ is the normal cumulative distribution function, *N *the multivariate normal distribution, *δ *the one-point distribution, and *W*^-1 ^the inverse Wishart distribution. *n*_*ρ *_denotes the number of patients in dataset *ρ*. **J**^*κ *^is the diagonal matrix with the non-zero elements in the vector  as diagonal elements. Similarly,  and  are the elements of **x**_*ρi *_and ***β***_*ρ*_, respectively, that correspond to the non-zero elements in  (**x**_*ρi *_relates to patient *i *in dataset *ρ *and ***β***_*ρ *_are the regression coefficients related to dataset *ρ*). The index *ρ *indicates that the coefficients varied by breast cancer study (*W, D, M, S, P*).

We assumed *a priori *that on average 100 genes should be included in the signature (i.e. that  = 100, where *E *denotes the expectation operator). This assumptions was based on the number of genes used in previously reported signatures [13-16]. Further, we assumed that the greater the number of times a cancer pathway regulated gene's name has been mentioned together with breast cancer in free, fulltext PubMed articles, the greater is the *a priori *scientific support that the gene's regulation is correlated with breast cancer relapse. Thus, the larger the *k*-value (as defined in (1)) associated with a given gene, the greater the *a priori *probability of the gene to be included in the signature. In order to give all genes a chance of being selected, we assign the *k*-value 1 to a gene not reported in NetPath. Genes reported in NetPath but not mentioned in any free PubMed article get the *k*-value 2. The prior distribution for  is founded on the idea that the probability of selecting *one *variable *j *of the 22283 variables is proportional to the variable *j*'s *k*-value, i.e. *Pr*(select *j *in one draw) = . Thus, the probability of *not *selecting the variable *j *is  and the probability of selecting variable *j *in *m *independent draws is . All genes thus have a chance to get selected and all genes have a chance of not being selected.

Apart from the prior distribution of , no prior knowledge was assumed for the remaining model parameters (i.e. noninformative priors was used for , Σ^*κ *^and *m*).

Model I was validated by fitting it five times, each time using three of the five datasets (**X**, **r**, ***γ***)_*ρ*_, *ρ *∈ {*W, D, M, S, P*}, as training sets and the remaining two as independent test sets. The benefit of using multiple datasets for fitting Model I was assessed by using the same partitioning of the five datasets; however this time only one of the three training sets was used for actually fitting the model (see Additional file 1, Table S1 for the partitions and results). The gain of exploiting the prior information was assessed by modifying Model I so that *p *= 100/22283 in (2), in which case all genes get an equal *a priori *probability to be included in the signature.

To afford comparisons with the gene signatures published in Wang *et al*. [13], Miller *et al*. [14], Sotiriou *et al*. [15], and Pawitan *et al*. [16], we modified Model I according to:(3)

where  denotes the subset of **x**_*ρi *_that corresponds to the gene signature *ρ*_*sig *_derived in publication *ρ*,  is the number of genes in the signature *ρ*_*sig*_, and  the identity matrix of rank .

To derive a gene signature using all the data, we fitted Model I as described in (2). We counted the number of times that each gene appeared in the 200,000 draws from the posterior distribution in the Markov chain Monte Carlo algorithm (after 200,000 draws burn-in, see below). We defined the appearance frequency of a gene *g*_*j *_as the number of appearances of *g*_*j *_divided by the total number of iterations (i.e., 200,000 here). We reasoned that the genes with the highest appearance frequencies play the strongest role in predicting the response. The top 100 genes, according to appearance frequency, were selected and are found in Additional file 1, Table S2.

#### Model II

We modeled the time to development of distant metastases according to:(4)

where the denotations are as in Model I, and *ε*_*ρi *_is the error term for patient *i *in dataset *ρ*. Model II was validated by fitting it five times, each time using three of the five datasets (**X**, **r**, ***γ***)_*ρ*_, *ρ *∈ {*W, D, M, S, P*}, as training sets and the remaining two as independent test sets.

### Proteomics data: Analyzing PDZ domain-peptide interactions

The peptides and the aligned PDZ sequences (Fig. 4) were, after removal of alignment positions containing gaps, converted to numerical vectors capturing the physicochemical properties of each sequence by using the tciz scales of Muthas *et al *[36]. The tciz scales are the amino acids' component scores in the two first principal components derived from a principal component analysis of physicochemical properties of 113 natural and non-natural amino acids. The two tciz scales thus code each sequence residue into two real-valued numbers, which are strongly correlated with the amino acids size and polarity [36]. The tciz scales thus allow us to numerically characterize peptides by concatenating the tciz numbers for all residues in the peptide. Similarly, we could numerically characterize an interaction between a PDZ domain and a peptide as the concatenated tciz description vectors of the PDZ domain and the peptide. Characterizing all interactions in the Stiffler *et al*. [22] and Chen *et al*. [23] datasets gave us a matrix, **X**, with 2306 observations (2019 from mouse, 147 from *C. elegans*, and 140 from *D. melanogaster *PDZ domains) and 154 variables (67 residue positions, *r*_1_,...,*r*_67_, in the alignment of the PDZ domains were "gap-free", and each position was coded with two tciz numbers: 67·2 = 134 variables; the peptides contained 10 residues each: 10·2 = 20 variables).

Let **H **= (**X**, *H*(**X**)) be mean centered and scaled to unit variance, where *H*(**X**) denotes the second order interaction terms of the variables in **X**. **H **thus has 2306 rows and 11935 columns. Let the column vector **y **= (*y*_1_,...,*y*_2306_) be the response variable, where *y*_*i *_= 1 represent that the PDZ domain and the ligand interact and *y*_*i *_= 0 represents that they do not interact. We assumed, with support from the literature [37] and the data from Stiffler *et al*. [22], that some PDZ domains are more promiscuous binders than others. This resulted in a correlated error structure when modeling the data. This is an assumption violation in standard generalized linear models, but could be accommodated for in our Bayesian setting by allowing parameter estimates to vary among PDZ domains. Thus, let *ϕ *= 1,...,96 denote the 96 PDZ domains in the data from Stiffler *et al*. [22] and Chen *et al*. [23] (82 from mouse, 7 from *C. elegans*, and 7 from *D. melanogaster*).

The PDZ domain residues *r*_1_,...,*r*_67 _and the peptide residues *p*_1_,...,*r*_10 _are associated with the integers *c*_1_,...,*c*_77_, where the *c*-values represent the number of contacts its corresponding residue makes with a ligand, as estimated by SAS by analyzing all solved 3D structures of PDZ domain-peptide complexes available in the Protein Data Bank [25] (PDB) (Fig. 4). We construct the row vectors(5)

where *H*(**c**_**X**_) denotes the second order interaction terms of **c**_**X**_. **c**_**X **_thus represents the *c*-values of the tciz-coded residues *r*_1_,...,*r*_67 _and *p*_1_,...,*p*_10 _incremented by 1. The *c*-values were incremented by 1 to ensure that all variables in **H **have a chance of being selected (i.e. including those with *c*-values equal to zero).

#### Model III

We modeled the interaction between PDZ domains and peptides using a Bayesian hierarchical probit model according to:(6)

where  is the *i*th row in  (the rows of **H **corresponding to PDZ domain *ϕ*). *d*_*j *_is the *j*th value in the row vector **c**_**X**, *H*(**X**)_. Hence, the *a priori *probability of a variable to be selected is larger, the larger the number of contacts the PDZ residue it is derived from makes with the ligand (the assumption being that the more contact a PDZ residue makes with the ligand, the greater our *a priori *belief that the residue is important for the interaction). The other denotations are analogous to the ones used in Model I. The prior for *m*, i.e. the parameter controlling the number of selected variables, was chosen so that roughly one twentieth of the total number of variables was selected (i.e. so that  = 11935/20, where *E *denotes the expectation operator). The mean of the prior distribution for *m *was estimated using cross-validation on the training data. Model III was also modified to assess the use of the prior information about which residues that are important for governing the interaction between PDZ domains and peptides. We then set *p *= 1/11935.

#### Residue coupling depicted in Fig. 5c

The coupling was estimated by studying the interaction terms in **H **formed between tciz scales from one residue in the PDZ domain and one in the peptide as outlined in Prusis *et al*. [38]. The figure displays the five such interaction terms with the largest (absolute-valued) modes of the regression coefficient's distributions. The network residues are color coded according to the physicochemical property that is estimated to be important for the network; residue size is shown in blue and residue polarity in red. Note that the same PDZ domain 3D structure (*α*1-syntrophin PDZ, PDB accession number 2PDZ) has been used in all three pictures since the structures of Lap4(2/4) and Lin7(1/1) from *C. elegans *and *D. melanogaster *have not been solved. The residue labels refer to the amino acid at the given position in Lap4(2/4) and Lin7(1/1) and do thus not correspond to the structure of the depicted amino acids. Residue numbering is according to 2PDZ.pdb.

### Bayesian P-values

To explain the Bayesian P-values used in the article, we give the following example: Let  be the two datasets that were removed when fitting Model I and  the remaining three datasets. Further, let ***β ***and ***β' ***be the regression coefficients when Model I was fitted with informative and noninformative priors, respectively. By sampling from the posterior distributions of ***β ***and ***β'***, we could estimate the distributions of  and . The Bayesian P-value could then be computed as . This idea is closely related to the method suggested in Xiao-Li [39].

### ROC curves

ROC curves are plots of the true-positive fraction (TPF) plotted as a function of the false-positive fraction (FPF) for a binary classifier system as its discrimination threshold is varied. The TPF and FPF are calculated as follows: TPF = TP/(TP + FN) and FPF = FP/(FP + TN), where TP = true positives, FP = false positives, FN = false negatives, and TN = true negatives. The larger the area under the curve (the AUC), the better the classifier. The AUC is equal to the Mann-Whitney U statistic [40], a non-parametric test for assessing whether two independent samples of observations come from the same distribution.

### Model fitting

The posterior distributions for Model I, II, and III are not possible to derive in closed form. The models were thus fitted using Markov chain Monte Carlo (MCMC) methods (see Robert and Casella [41] for an excellent account on MCMC methods). The MCMC algorithms were implemented in the statistical programming language R [42]. Three chains were run for each model and after a burn-in period of 200,000 samples, we collected the following 200,000 samples from the posterior distributions. Convergence was checked using the potential scale reduction factor [43]. The computations were performed on UPPMAX computational resources http://www.uppmax.uu.se/ under the project p2006026.

## Authors' contributions

ME devised and implemented the proposed eScience-Bayes framework. OS was involved in program design and aided with the implementation. JESW supervised the project. All authors read and approved the final manuscript.

## Supplementary Material

Additional file 1 **Additional results**. Additional results obtained with Model I, II, and III. Specific information regarding the results are available within the additional pdf file. Click here for file
