# Supplementary material for: An eScience-Bayes strategy for analyzing omics data
Source: BMC Bioinformatics. 2010 May 26;11:282. doi: 10.1186/1471-2105-11-282 (PMC2887810; doi:10.1186/1471-2105-11-282)
Supplement: Additional file 1 — Additional results. Additional results obtained with Model I, II, and III. Specific information regarding the results are available within the additional pdf file. [file 1471-2105-11-282-S1.PDF]

# An eScience-Bayes strategy for analyzing omics data

## Supplementary material

In the supplementary material we use the following short-hand denotations:

- W - $(\mathbf{X}, \mathbf{r}, \boldsymbol{\gamma})_W$
- D - $(\mathbf{X}, \mathbf{r}, \boldsymbol{\gamma})_D$
- M - $(\mathbf{X}, \mathbf{r}, \boldsymbol{\gamma})_M$
- S - $(\mathbf{X}, \mathbf{r}, \boldsymbol{\gamma})_S$
- P - $(\mathbf{X}, \mathbf{r}, \boldsymbol{\gamma})_P$

## References


[1] Wang, Y. *et al.* Gene-expression profiles to predict distant metastasis of lymph-node-negative primary breast cancer. *Lancet* **365**(9460), 671–679 (2005).

[2] Miller, L.D. *et al.* An expression signature for p53 status in human breast cancer predicts mutation status, transcriptional effects, and patient survival. *Proc. Natl. Acad. Sci. USA* **102**(38), 13550–13555 (2005).

[3] Sotiriou, C. *et al.* Gene expression profiling in breast cancer: understanding the molecular basis of histologic grade to improve prognosis. *J. Natl. Cancer Inst.* **98**(4), 262–272 (2006).

[4] Pawitan, Y. *et al.* Gene expression profiling spares early breast cancer patients from adjuvant therapy: derived and validated in two population-based cohorts. *Breast Cancer Res.* **7**(6), R953–64 (2005).

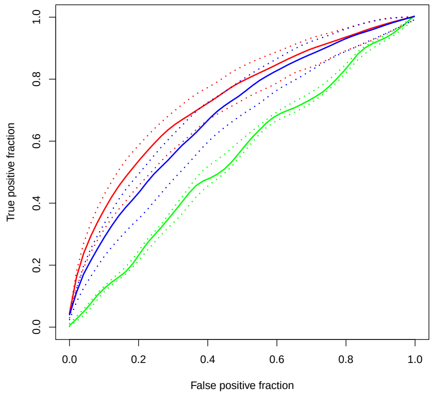


(a) Trained on D, S, P (multiple), and D (single). Prediction of observations in W and M.

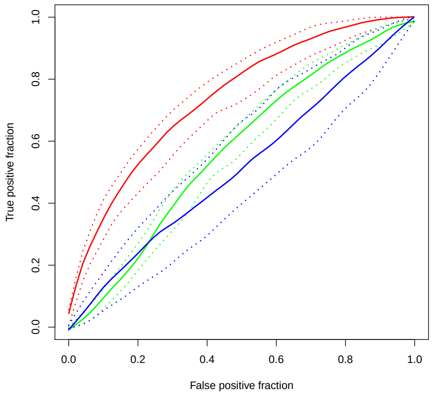


(b) Trained on W, S, P (multiple), and P (single). Prediction of observations in D and M.

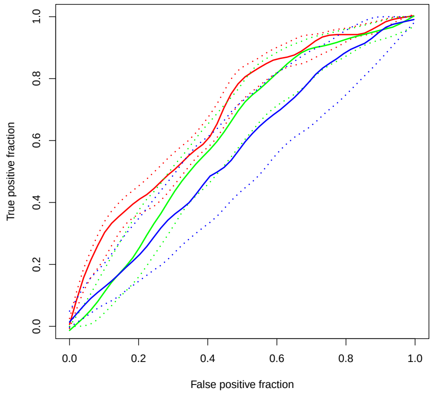


(c) Trained on W, M, P (multiple), and W (single). Prediction of observations in D and S.

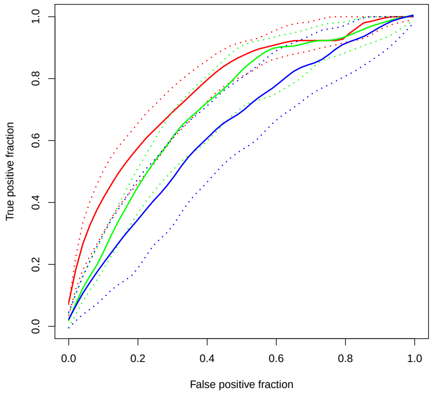


(d) Trained on W, D, M (multiple), and W (single). Prediction of observations in P and S.

Figure 1: ROC curves from training Model I on different datasets. Red lines show results when prior information was used, green when no prior information was used, and blue when only a single dataset was used for training. The solid lines show the mode of the ROC curve distributions, dotted lines show 95% Bayesian confidence intervals. AUC-values for the ROC curves are given in Supplementary Table 1.

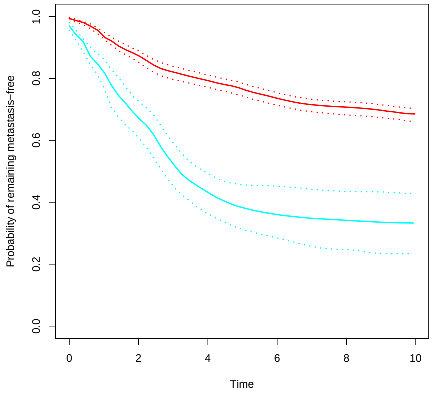


(a) Trained on D, S, P. Prediction of observations in W and M.

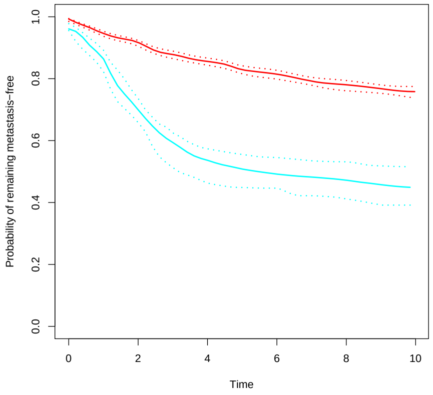


(b) Trained on W, S, P. Prediction of observations in D and M.

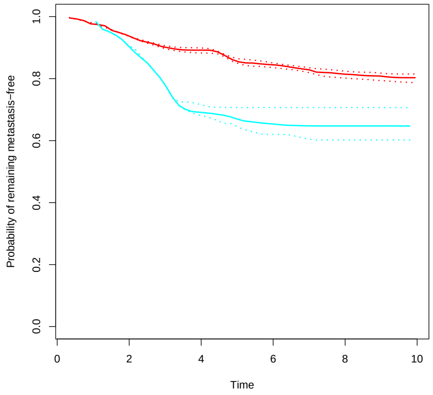


(c) Trained on W, M, P. Prediction of observations in D and S.

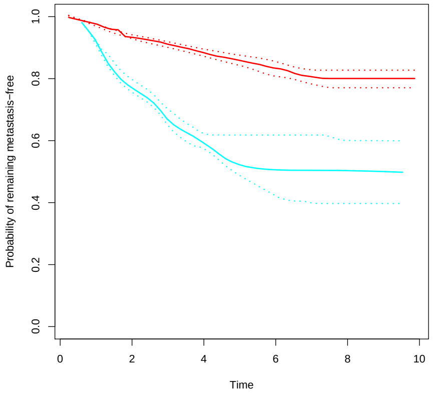


(d) Trained on W, D, M. Prediction of observations in P and S.

Figure 2: Kaplan-Meier curves for patients predicted by Model I (at 80% specificity) to develop distant metastases before (red) and after (cyan) 5 years. Dotted lines show 95% Bayesian confidence intervals.

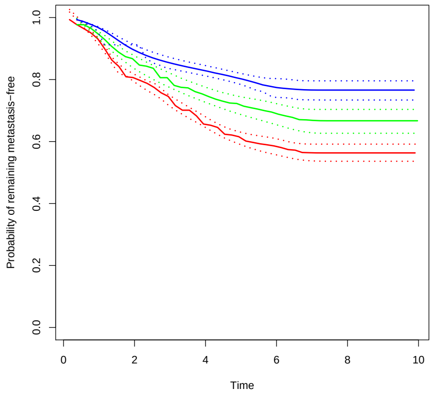


(a) Trained on D, S, P. Prediction of observations in W and M.

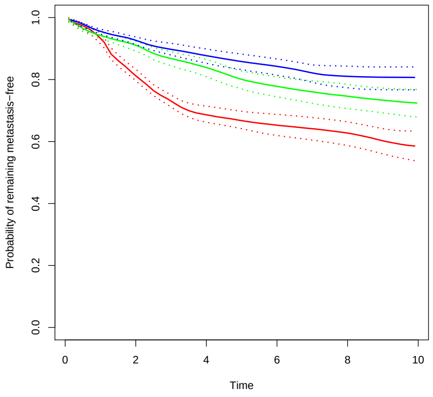


(b) Trained on W, S, P. Prediction of observations in D and M.

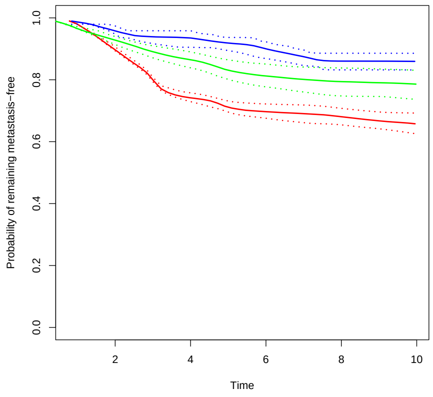


(c) Trained on W, M, P. Prediction of observations in D and S.

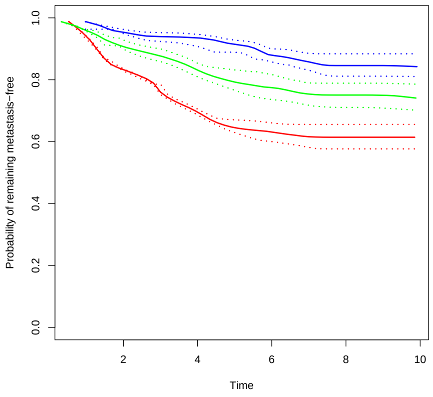


(d) Trained on W, D, M. Prediction of observations in P and S.

Figure 3: Patient survival times predicted by Model II. The figure shows the Kaplan-Meier curves for the patients belonging to the predicted percentiles 0-33, 34-66, 67-100 (red, green, and blue curves, respectively). Dotted lines show 95% Bayesian confidence intervals.

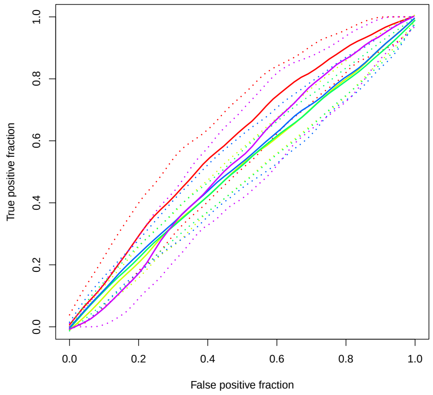


(a) Signature from Wang *et al.*

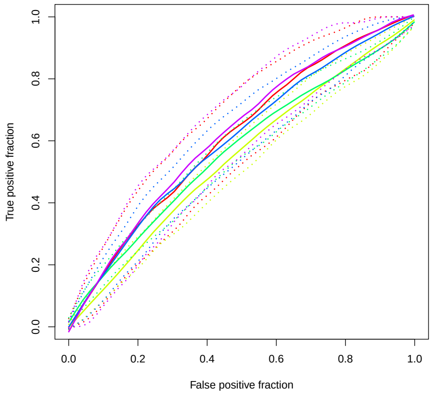


(b) Signature from Miller *et al.*

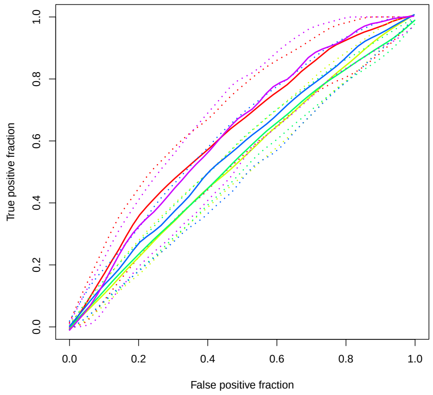


(c) Signature from Sotiriou *et al.*

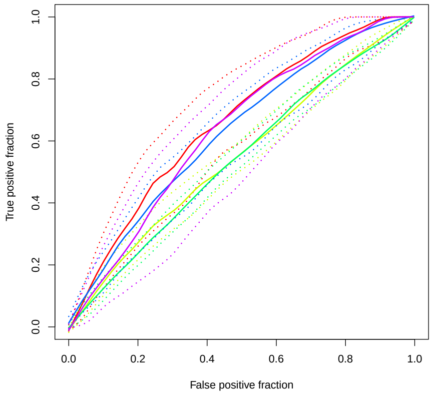


(d) Signature from Pawitan *et al.*

Figure 4: ROC curves from Model I trained using the gene signatures published in Wang *et al.*[1], Miller *et al*[2], Sotiriou *et al*[3], and Pawitan *et al*[4]. Red curve - W, D, M used as training sets, and S, P as test sets. Green curve - D, S, P used as training sets, and W, M as test sets. Purple curve - W, M, P used as training sets, and D, S as test sets. Blue curve - W, S, P used as training sets, and D, M as as test sets. Yellow curve - D, M, S used as training sets, and W, P as test sets. Dotted lines show 95% Bayesian confidence intervals.

| | $AUC_{W,M}$ | $AUC_{W,P}$ | $AUC_{D,M}$ | $AUC_{D,S}$ | $AUC_{S,P}$ |
|---|---|---|---|---|---|
| Prior | 0.73 (0.68; 0.77) | 0.76 (0.71; 0.80) | 0.74 (0.68; 0.77) | 0.68 (0.65; 0.71) | 0.77 (0.72; 0.82) |
| No prior | 0.55 (0.54; 0.56) | 0.57 (0.55; 0.58) | 0.57 (0.55; 0.59) | 0.61 (0.56; 0.67) | 0.71 (0.62; 0.76) |
| Single | 0.69 (0.62; 0.73) D | 0.51 (0.44; 0.60) M | 0.51 (0.39; 0.62) P | 0.56 (0.45; 0.65) S | 0.64 (0.54; 0.71) W |
| W sig. | 0.51 (0.48; 0.54) | 0.51 (0.48; 0.55) | 0.52 (0.46; 0.57) | 0.53 (0.43; 0.62) | 0.61 (0.51; 0.68) |
| M sig. | 0.58 (0.53; 0.61) | 0.55 (0.49; 0.58) | 0.61 (0.52; 0.65) | 0.64 (0.54; 0.70) | 0.63 (0.51; 0.71) |
| S sig. | 0.53 (0.50; 0.57) | 0.55 (0.49; 0.60) | 0.56 (0.48; 0.64) | 0.63 (0.52; 0.70) | 0.65 (0.55; 0.73) |
| P sig. | 0.56 (0.51; 0.57) | 0.57 (0.51; 0.59) | 0.63 (0.52; 0.70) | 0.65 (0.49; 0.73) | 0.68 (0.59; 0.77) |

Table 1: Area under ROC curve (AUC) for prediction of development of metastases within five years using Model I trained and tested on different datasets. The AUC is equal to the value of the Mann-Whitney U statistic, i.e. the higher the AUC value the better the performance of the prediction. The values are the mode of the distribution of AUC in each case, and the values in the parentheses are the lower and upper limit of the 95% Bayesian confidence interval. We regard two AUC values to be significantly different on the 0.05-level if the confidence interval of one value does not overlap the mode of the other. We used three of the five datasets for training and the remaining two for testing, except in the row named 'single', where only a single dataset was used for training. The denotations are as follows: $AUC_{\eta,\upsilon}$ - the area under the ROC curve is calculated from predicting patients in dataset $\eta$ and $\upsilon$, Prior - prior information retrieved using the Web services NetPath, DictService, and Entrez utilities was used in the modeling, No prior - the retrieved prior information was not used, Single - only a single dataset was used for training (the letter after the AUC values shows which dataset was used for training), W sig. - The genes from the signature derived in Wang *et al.*[1]

| **Relevant *a priori* and *a posteriori*** | **Nonrelevant *a priori* but relevant *a posteriori*** |
|---|---|
| 5743, 545, 5595, 6462, 1029, 133, 1543, 2885 ,1545, 3558, 2810, 1030, 1081, 10395, 840, 4335, 5047, 1028, 2078, 860, 2919, 8838, 6469, 7465, 6608, 8312, 3371, 4842, 5272, 1487, 2938, 6615, 1875, 6781, 5931, 2115, 182, 6839, 5055, 2297, 3909, 10550, 1613, 8643, 4289, 8572, 11077, 22937, 1435, 7052, 2587, 1746, 6691, 80324, 10207, 10282, 2869, 5908, 51693, 2959, 3949, 2257, 3781, 1002, 123, 55741 | 836, 27183, 10865, 9672, 983, 1734, 10036, 10005, 54552, 6540, 4775, 9863, 26333, 1981, 9212, 4502, 112950, 9456, 390998, 647288, 4848, 29105, 116496, 64423, 29103, 11284, 11178, 57597, 9245, 54558, 64694, 645644, 56926 |

Table 2: Top 100 genes selected using all five datasets W, D, M, S, and P. The table shows Entrez gene id:s.
